# A Uniquely Targeted, Mobile App-Based HIV Prevention Intervention for Young Transgender Women: Adaptation and Usability Study

**DOI:** 10.2196/21839

**Published:** 2021-03-31

**Authors:** Lisa M Kuhns, Jane Hereth, Robert Garofalo, Marco Hidalgo, Amy K Johnson, Rebecca Schnall, Sari L Reisner, Marvin Belzer, Matthew J Mimiaga

**Affiliations:** 1 Ann & Robert H Lurie Children's Hospital of Chicago Potocsnak Family Division of Adolescent and Young Adult Medicine Chicago, IL United States; 2 Department of Pediatrics Feinberg School of Medicine Northwestern University Chicago, IL United States; 3 Helen Bader School of Social Welfare University of Wisconsin-Milwaukee Milwaukee, WI United States; 4 Division of Adolescent and Young Adult Medicine Children’s Hospital Los Angeles, University of Southern California Los Angeles, CA United States; 5 School of Nursing Columbia University New York, NY United States; 6 Fenway Institute Boston, MA United States; 7 Division of General Pediatrics Boston Children’s Hospital, Harvard Medical School Boston, MA United States; 8 Department of Epidemiology Harvard T H Chan School of Public Health Harvard University Boston, MA United States; 9 Center for Health Equity Research Brown University Providence, RI United States; 10 Department of Psychiatry & Human Behavior Alpert Medical School Brown University Providence, RI United States

**Keywords:** transgender persons, HIV, mobile app, mHealth, mobile phone

## Abstract

**Background:**

Young transgender women (YTW) are a key population for HIV-related risk reduction, yet very few interventions have been developed to meet their needs. Mobile health interventions with the potential for both efficacy and wide reach are a promising strategy to reduce HIV risk among YTW.

**Objective:**

This study aims to adapt an efficacious group-based intervention to a mobile app, Project LifeSkills, to reduce HIV risk among YTW, and to test its acceptability and usability.

**Methods:**

The group-based intervention was adapted to a mobile app, LifeSkills Mobile, with input from an expert advisory group and feedback from YTW collected during user-centered design sessions. A beta version of the app was then tested in a usability evaluation using a think-aloud protocol with debriefing interviews, recordings of screen activity, and assessments of usability via the Post-Study System Usability Questionnaire (PSSUQ) and the Health Information Technology Usability Evaluation Scale (Health-ITUES).

**Results:**

YTW (n=8; age: mean 24 years, SD 3 years; racial or ethnic minority: 7/8, 88%) provided feedback on the app prototype in design sessions and then tested a beta version of the app in a usability trial (n=10; age: mean 24 years, SD 3 years; racial or ethnic minority: 8/10, 80%). Both usability ratings (Health-ITUES: mean 4.59, SD 0.86; scale range: 1-5) and ratings for satisfaction and accessibility (PSSUQ: mean 4.64, SD 0.90; scale range 1-5) were in the good to excellent range. No functional bugs were identified, and all mobile activities were deployed as expected. Participant feedback from the usability interviews indicated very good salience of the intervention content among the focal population. Participants’ suggestions to further increase app engagement included adding animation, adding audio, and reducing the amount text.

**Conclusions:**

We conclude that the LifeSkills Mobile app is a highly usable and engaging mobile app for HIV prevention among YTW.

## Introduction

### Background

HIV prevalence is disproportionately high among transgender women. Meta-analyses of previous studies indicate that the prevalence of laboratory-confirmed HIV infection among transgender women in the United States is between 19% and 28% [1,2], a level that is more than 30 times the odds of HIV-infection among the general population of adults of reproductive age. Younger and racial minority transgender women are particularly susceptible. A recent HIV testing initiative that involved over 1800 transgender women from 23 cities found the highest percentage of confirmed HIV seropositivity among Black transgender women (prevalence ratio: 3.13; 95% CI 1.45-6.78) [3]. In a previous study analyzing data from the local testing of over 500 transgender women (with no known previous positive HIV test results) in Miami, San Francisco, and Los Angeles, the highest number of cases was found among young transgender women (YTW) aged between 20 and 29 years (ie, 45%) [4].

Despite the high rates of HIV infection among transgender women, there are few interventions that have been developed specifically to address their underlying mechanisms of sexual risk [5]; only 2 interventions of sufficient scientific rigor and quality are included in the Center for Disease Control and Prevention’s Compendium of HIV prevention interventions [6]. These two interventions include our LifeSkills group–based HIV prevention intervention for YTW aged between 16 and 29 years [7] and the Couples HIV Intervention Program, a face-to-face counseling intervention for adult transgender women and their partners [8]. Given the prevalence of HIV infection among YTW and the dearth of evidence-based interventions, there is a need to continue to develop and test HIV prevention interventions for YTW that are both efficacious and scalable.

### Development and Testing of the LifeSkills Intervention

The LifeSkills intervention addresses the specific structural, developmental, and interpersonal challenges of HIV prevention among YTW [9,10]. It is based on empowerment theory [11-14] and was developed using a community-engaged approach to address the everyday social and structural drivers of risk among YTW [15-18]. The intervention promotes HIV-related information (education), motivation to reduce risk, and behavioral skills, consistent with the Information-Motivation-Behavioral skills model, one of the most parsimonious models of HIV-related behavior change [19-23], with the greatest utility for those at very high HIV risk [24].

The LifeSkills intervention was tested in a 2-city randomized controlled trial (RCT) and found to be efficacious with high rates of satisfaction. The RCT was conducted between 2012 and 2016 among 190 sexually active YTW [25]. The LifeSkills group had a 39.8% greater mean reduction in condomless sex acts (vaginal or anal) at 12-month follow-up compared with preventive care arm (0.71 vs 1.40; risk ratio: 0.60; 95% CI 0.50-0.72; *P*<.001) [7]. In addition, participants in the intervention arm reported high satisfaction with the curriculum: 98% of participants indicated they would refer a friend to receive LifeSkills [7]. However, some participants reported barriers to the uptake of the intervention, including problems attending group-based sessions because of competing priorities, difficult travel from other areas of the city or suburban locations, and scheduling conflicts. In addition, some participants expressed safety concerns regarding frequent travel to the study site, not because the site locations were unsafe but because travel on public transportation is often unsafe for YTW [26]. Furthermore, similar to group-based supportive therapy, a group-based HIV risk reduction intervention is not suitable for all who might benefit from it because of shyness, personality clashes, and the perception of lack of confidentiality.

Given these findings, we sought to extend the potential reach, accessibility, and privacy of the intervention via adaptation to a mobile app. Mobile health (mHealth) approaches (eg, health intervention via smartphones, tablet computers, or other mobile devices) for HIV prevention have the advantage of simple interface for users, accessibility anywhere internet access is available, and relative affordability and have been promoted specifically to reach stigmatized and disenfranchised populations [27,28]. A review of 62 HIV-specific mHealth studies found that most target HIV-positive adults (45%) and feature alerts and reminders (60%) as a primary health promotion strategy [27]. The findings suggest the potential for positive effects on health promotion across the HIV care continuum [27,28]. Although no HIV studies have specifically focused on HIV prevention among YTW, in a review of evidence for another key population, men who have sex with men, Schnall et al [29] found evidence that mHealth approaches, particularly web-based videos and education modules, show significant effects for both the reduction of HIV risk behavior and promotion of HIV testing. They also found that few mHealth approaches focus on the most recent biomedical prevention strategies, such as pre-exposure prophylaxis (PrEP), postexposure prophylaxis (PEP), and Treatment as Prevention (TasP). More specifically, evidence suggests that web-based interactive and educational approaches among youth aged between 13 and 29 years [30,31] are efficacious for delaying sexual initiation and increasing knowledge of HIV and sexually transmitted infections and condom self-efficacy [32]. In addition, gaming components of HIV-specific mHealth interventions are becoming increasingly important, particularly among youth, because they are designed to be fun and may increase interaction and engagement, creating opportunities for learning [33].

This study aims to adapt the LifeSkills group–based intervention to a mobile platform and test its usability in the focal population, YTW aged between 16 and 29 years.

## Methods

### Overview

Extensive formative work to develop the LifeSkills intervention content was completed together with YTW [9]. Thus, our approach in this study was to adapt the original content to a mobile app. The adaptation of the LifeSkills Mobile app is based on best practices for the adaptation of evidence-based interventions and user-centered design [9,34]. Although focused primarily on new populations or settings, recommended adaptation strategies for evidence-based HIV prevention interventions include iterative processes, multiple methods and informants, and the preservation of core intervention components [35-37]. Thus, our adaptation process sought to preserve the components of the original intervention (ie, empowerment, information, motivation, and behavioral skill building), add biomedical prevention content (PrEP and TasP), and maintain its *look and feel* (eg, stories and scenarios of YTW) and unifying themes (ie, *House of LifeSkills*), while using an iterative, multimethod process with input from various key sources.

In this study, we adapted the in-person, group-based LifeSkills to mobile app and tested it in 3 phases: (1) prototype development, (2) design sessions and app development, and (3) usability testing (Multimedia Appendix 1).

The institutional review board at Ann & Robert H. Lurie Children’s Hospital of Chicago approved all study procedures, and written consent was obtained from all study participants.

### Prototype Development

In the first phase of the adaptation process, we translated the group-based intervention curriculum, which consisted of 6 sessions (4-5 activities each), into a paper-and-pencil app prototype with input from our group of 6 investigators and the study coordinator, who have extensive expertise in transgender health, youth-centered intervention development, HIV prevention and related behavioral intervention development efforts, and app development and informatics. In addition, 2 former facilitators of the group-based LifeSkills intervention served as community consultants to the project to inform the translation of the behavioral activation components (eg, activities, games, and scenarios) and *look and feel* to mobile app format. The development of the prototype occurred iteratively, with each draft reviewed in monthly meetings with the advisory group. The primary focus of prototype development was to maintain the core themes and activities of the original intervention, to draft interactive mobile activities to replace group-based activities, and to create engaging characters (to replace group facilitators). The end product was a paper-and-pencil prototype, which served as a blueprint of content for building the app.

### Design Sessions and App Development

The draft paper-and-pencil prototype was provided to our software collaborator, One Cow Standing, as the first step in the app-building process. Wireframes were developed by One Cow Standing based on the prototype and the draft images of app characters and scenarios.

The draft prototype and images were then presented to the end users during the design sessions. Participants were identified and recruited from a registry of participants in previous studies. Individuals were eligible for design sessions based on (1) age between 16 and 29 years, (2) self-identity as transfeminine, (3) a history of condomless anal or vaginal sex, and (4) English speaking. We divided the prototype content into 3 subsections (of approximately equal length) and asked participants to review one-third of the content activity-by-activity, such that each subsection was reviewed by at least two participants. For each activity, we asked participants a set of semistructured questions focusing on user-centered aspects of adaptation to mobile, for example, whether they found the mobile app activities engaging (if so, why, and if not, why not), whether they understood the activity content (ie, wording, phrasing, and instructions), if they found it effective (ie, did it raise your awareness, motivate you, and introduce new skills), perceptions of the images and graphics, coverage of topics (ie, missed anything important), and any additional feedback or suggestions. Feedback from these sessions was recorded in extensive written notes for analysis. Data were rapidly analyzed for key actionable themes (eg, content to remove, revise, and add) [38], which were translated to modifications in the prototype.

We then implemented the app build in a series of design sprints over a 5-month period. Design sprints are time-constrained, phased periods in which specific programming work is completed, reviewed, and revised. In each sprint, programming of content was followed by testing of functional components and review of graphics and images by an abbreviated team of 4 alpha testers (LK, JH, and 2 community consultants), followed by discussion in weekly meetings with One Cow Standing. This process was facilitated by overlaying the test version of the app with BugHerd software (Splitrock Studio) [39], which was used to pinpoint functional bugs and areas in need of design improvements or modifications, with items placed in action queues to track completion. The final review was conducted by the entire advisory group of investigators in the last sprint with feedback and revision. The end product of this phase was a beta version of the app ready for usability testing.

### Usability Testing

We performed usability testing on the beta version of the app to identify violations of usability principles and any potential obstacles to use. Eligibility for this phase of testing was the same as for design sessions; individuals who had participated in design sessions could also participate in usability testing, which provided an opportunity for feedback on the execution of design suggestions. Usability testing is an iterative process that involves testing the app and then using results to change the app to better meet users’ needs. The usability testing phase was guided by the Health Information Technology Usability Evaluation Model (Health-ITUEM) to evaluate mHealth technology [40], which includes a focus on quality, perceived usefulness, perceived ease of use, and user control.

We planned to execute at least two rounds of usability testing to identify potential problems and then correct and test the app, with rounds of testing added as needed to resolve all issues. Through a think-aloud protocol [41], each participant was asked to perform tasks that closely mirror the intended end use of the app. We used Morae software (TechSmith Corporation) [42] to record the interview audio and screenshots of the problem areas.

Participants completed a brief demographic and technology use survey and then rated the app’s usability using validated instruments, including the Health Information Technology Usability Evaluation Scale (Health-ITUES) [43,44] and Post-Study System Usability Questionnaire (PSSUQ) [45]. The Health-ITUES evaluates tasks or expectations relevant to our usability dimensions, including impact, usefulness, ease of use, and user control. The PSSUQ measures accessibility and satisfaction. Both scales were rated on a 1- to 5-point scale, with higher scores indicating higher usability. We also measured satisfaction using the Client Satisfaction Questionnaire (CSQ) [46], which includes questions on overall quality and expectations. We measured acceptability with questions related to the use of language and the appeal of activities and artwork. These ratings were analyzed using measures of central tendency and dispersion.

### Qualitative Data Collection and Analysis

At the end of the think-aloud session, we asked for additional open-ended feedback on perceptions of the app in a brief, semistructured interview. Interview data were analyzed using a thematic analysis approach [47,48] to identify key themes related to our usability dimensions, particularly perceptions of information quality, intention to use the app, satisfaction with the content, and potential impact of HIV risk reduction.

## Results

### Prototype Development

The original LifeSkills intervention consists of 6 group-based sessions (4-5 activities each), which were shortened and consolidated and then translated to a paper-and-pencil prototype consisting of the following 4 app-based modules: module 1: TransPride; module 2: Breaking Down Barriers; module 3: Educating Yourself, and module 4: Protecting Yourself. For the mobile app, we consolidated 2 modules in the original intervention (*communication and respect*, which introduces assertiveness skills training, and *skill building*, which applies these skills) into 1 mobile module, *Breaking Down Barriers* (module 2), which includes both training and application and eliminates some redundancy in the original intervention. Table 1 lists the general themes and intervention targets reflected in each module and the associated theoretical constructs. In addition, a unifying concept used in the original intervention, *The House of LifeSkills*, was maintained in the mobile version. This theme incorporates the language and symbols of the urban house and ball communities. The house and ball communities developed organically among largely gay, transgender, and gender-nonconforming youth of color in response to homelessness and family rejection, as a means of support as well as self-expression in the form of dance and other forms of performance art. The house and ball communities provide alternative family support in the face of the marginalization experienced by many urban transwomen [49,50]. In the LifeSkills intervention, the house and ball communities are a point of reference, as an example of the role of mentorship, community, and friendship within the transgender community. Symbols and language are used (sparingly) to contextualize the LifeSkills intervention as providing similar mentorship. All the primary themes, intervention targets, and theoretical constructs from the original LifeSkills intervention were maintained in LifeSkills Mobile.

**Table 1:** LifeSkills Mobile themes, intervention targets, and constructs.

Module	Theme	Intervention target	Theoretical constructs
1	TransPride	Self-concept, self-esteem, and sense of self in the context of societal marginalization	Empowerment and motivation
2	Breaking Down Barriers	Communication in the context of discrimination and violence	Skill building (effective communication and gaining access to resources)
3	Educating Yourself	Risks and benefits associated with sexual behavior and gender transition	Information (HIV and sexually transmitted infection risk and injection risk) and skill building (condom and needle use)
4	Protecting Yourself	Social context, experiences of abuse and violence, sexual risk, and substance use	Motivation (attitudes, norms, and intentions for safer sex) and behavioral skills (discussing sex and condoms with sexual partners and negotiating sexual safety, the context of drug and alcohol use, and sex work)

### Design Sessions and App Development

#### Design Sessions

The prototype was reviewed in design sessions by 8 members of the target population of YTW (age: mean 24 years, SD 3 years; range 21-28 years; racial or ethnic minority: 7/8, 88%). The sessions lasted 75 minutes on average. User-specific themes, including engagement, understanding, effectiveness, perceptions of the images and graphics, and coverage of topics, are presented in detail below, with representative comments from participants.

With regard to engagement, participants were largely in agreement that the gaming approach with stickers and trophies was fun, with one participant, a 24-year-old Black woman (D1), noting that they motivated her to keep going through the activities. In terms of understanding the content, participants described scenes and scenarios as realistic, relatable, and accurate. To improve understanding, participants suggested the use of *street* names for some of the illicit drugs in addition to the use of formal names (eg, *T* and *Tina* for methamphetamine and *leaf* and *weed* for marijuana). In terms of perceived effectiveness, participants commented that the salience and resonance of the content increased its effectiveness. For example, a 21-year-old woman who identified as gender nonconforming (D7) noted that the focus on content related to hormone use and other transition-related treatments would address common misconceptions. In addition, two participants, a 27-year-old multiracial woman (D5) and a 21-year-old White woman (D8), recommended that indicators of unhealthy relationships (in addition to the content on indicators of healthy relationships) be added because of the direct threat they represent to some transgender women (eg, abuse and physical violence) to increase its effectiveness. The perceptions of images and graphics were largely positive. Participants liked the images of characters and the artwork. For example, participants had mostly positive comments about the inclusiveness of gender expression depicted among characters (eg, early transition, gender variant, and feminine). In terms of coverage of topics, participants had positive comments about the addition of information on PrEP and PEP, noting that basic and accurate information on these topics is needed. In terms of additional comments and suggestions, one recurring theme was the perception that the app would improve the accessibility of the intervention to the target population of YTW. For example, a 25-year-old Black woman (D3) stated that the app “would be good for those who might not be comfortable coming to a group...Now I’m looking at a screen, I can go back to it...It’s going to be dope.”

A 21-year-old multiracial woman (D4) shared that the app would reach young women who did not know about the original LifeSkills groups or other resources. She stated:

Everyone is on their phone. So [the app] can reach people that didn’t even know that any of this was going on.

Overall, the findings from this phase suggested successful adaptation of the content to the mobile prototype with some suggestions for improved language and additions to content. The prototype was updated with these changes to inform the full development of the alpha and beta versions of the app.

#### Design Sprints

LifeSkills Mobile is a web app developed for mobile use (ie, not a native app intended for distribution through app stores). The decision to develop a web app was based on the higher cost of developing native apps and the additional complications posed by native apps, including distribution rules through app stores that change frequently. A web app is cross-platform and does not require platform-specific modifications, thereby increasing flexibility. Although browsers cannot use all the native features of a mobile device, they can present rich and engaging content and use device features, such as the camera (which is used in module 1). There are also no design elements in LifeSkills Mobile that require native app features. Finally, because it is not platform specific, it can reach a wider audience as all modern mobile devices have web browsers and are not limited to mobile phones for use; it can be accessed via a tablet or desktop computer.

The LifeSkills Mobile intervention was designed using JavaScript as the underlying programming language for both the client and server portions of the app. JavaScript is a cross-platform language that runs on all major operating systems. It is also the de facto programming language of the World Wide Web. This allows the code to be uniform and consistent as well as to easily make updates and modifications. The server component consists of a representational state transfer app program interface developed in Node.js (OpenJS Foundation) [51] using the Express framework. The database is MongoDB (MongoDB Inc) [52]. The web client interface was developed using the Vue.js (Vue.js) [53] framework.

Table 2 lists the final set of activities associated with each module in the beta version of the app. Module 1: TransPride sets the tone for the entire intervention and is devoted to empowerment themes. It includes transgender-specific success stories and a structured activity to create a participant’s own aspirational story (Icon Promo) and finally a goal setting activity that is updated throughout the intervention. Module 2 focuses on effective communication to meet basic needs (Communication Styles) and applies it to a scenario of escalating violence (Assertiveness Under Pressure), an activity to deconstruct and activate basic aspects of communication (both verbal and body expression), and a second scenario in a clinical care setting in the context of transspecific discrimination (Health care Barriers to create awareness of situations, triggers, feelings, and actions). Similarly, module 3 includes activities to raise awareness of sexual, injection, and other risk and practice correct steps for condom and injection use. Module 3 also includes information and resources specific to PEP and PrEP as well as HIV transmission, HIV care, medication adherence, and TasP. In module 4, specific contexts of risk are addressed, including relationship contexts (eg, power dynamics), challenges to skill building (eg, disclosure, substance use, commercial sex work, and partner selection and negotiation), and ending with an action plan to activate goals set and reviewed throughout the intervention.

The intervention activities, which consisted of scenarios, educational material and games, and role plays, were adapted to a mobile format using automated responses, drop-down menus, comics, and gamification to engage participants. For example, the app allows the user to input their own goals via drop-down menus, which differ based on personal experience and circumstances; these goals are challenged throughout the intervention by scenarios that differ between individuals (eg, substance use and involvement in sex work). Gamification is the use of game-like rewards and incentives to increase motivation and sustain intervention engagement over time [54]. We used two elements of gamification: (1) educational games and (2) persuasive games [55]. Education games are small games or minigames that use elements such as drag-and-drop, speed, and memory. Simulation was used for role-playing, in which participants had to envision themselves in different roles and choose the correct actions and dialogs to solve a task [56]. Persuasive games are used to motivate participants; participants have a tally bar (Multimedia Appendix 2) and supportive messages to motivate them to complete LifeSkills. Participation is incentivized with stickers for each activity and trophies for completion of modules. The YTW characters represent diverse gender presentations, racial or ethnic backgrounds, and HIV statuses. Similar to the group-based version of LifeSkills, these characters present scenarios to open each session and motivate participation in the next session.

**Table 2:** LifeSkills Mobile app modules and activities.

Module	Activity number	Activity title
Welcome	1	Welcome to LifeSkills Mobile
TransPride	2	Icon Promo (Pride)
TransPride	3	Why LifeSkills? (Risk and Protection)
TransPride	4	Get Your Life! (Setting Goals)
Breaking Down Barriers	5	Communication Styles
Breaking Down Barriers	6	Assertiveness Under Pressure
Breaking Down Barriers	7	Health care Barriers
Educating Yourself	8	Jeopar-T (Transition)
Educating Yourself	9	HIV True False
Educating Yourself	10	Bucket List! (Low, Medium, High Sex Risk)
Educating Yourself	11	Condom Use Steps
Educating Yourself	12	Safer Injection Steps
Protecting Yourself	13	Healthy Relationships
Protecting Yourself	14	Disclosure
Protecting Yourself	15	What are Your Boundaries
Protecting Yourself	16	In The Mix (Alcohol, Drugs)
Protecting Yourself	17	Sex Work and One Night Stands
Protecting Yourself	18	Setting Good Boundaries
Protecting Yourself	19	Red Flag Green Flag (Partner Negotiation and Selection)
Protecting Yourself	20	Action Plan (Accomplishing Goals)

### Usability Testing

The beta version of the app was completed, and usability evaluation was conducted among end users (n=10; age: mean 24 years, SD 3 years; range 21-28 years; racial or ethnic minority: 8/10, 80%; ever traded sex: 6/10, 60%; ever homeless: 10/10, 100%; General Educational Development/high school diploma or less: 7/10, 70%). Of 10 participants, 7 completed the design sessions. Of 10 participants, 9 indicated they use the internet *almost constantly*, and the same number (9/10) participants reported using the internet via smartphone or handheld device. A total of 5 participants completed the first round of usability testing, followed by an additional 5 participants who completed the second round of testing.

Usability ratings were in the good to excellent range (Health-ITUES: mean 4.59, SD 0.86), including on subscales for impact, usefulness, ease of use, and user control. Satisfaction and accessibility on the PSSUQ was also strong (mean 4.63, SD 0.90; range 1-5). On the CSQ, half of the participants rated the overall quality of the app as *good* and half as *excellent*. In total, 9 of the 10 participants indicated that they would *definitely* refer a friend to use the app.

No functional bugs were identified in either round of testing, and all activities were deployed as expected. Small errors in grammar, language, or image placement were identified by participants in the first round of testing, which were corrected for redeployment in the next iteration of usability testing. In addition, although there were no functional bugs identified, 2 activities, activity number 10 (Bucket List!) and number 17 (Sex Work and One Night Stands), caused confusion during completion in round 1 of testing. For activity number 10, items describing sexual acts are categorized by participants as *low*, *medium*, or *high risk*; however, participants reported confusion about how to categorize some risk behaviors. Brief definitions of each risk category were added for clarification. For activity number 17, strategies to protect oneself in contexts of commercial sex work are matched to risk contexts; however, participants expressed difficulty making matches because of perceived overlap in strategies. For this activity, redundancy in strategies was eliminated, and language was edited for clarity. With these changes, the intervention was redeployed with subsequent participants in round 2 of testing, with no further reports of problems understanding and completing the activities.

### Qualitative Findings

The analysis of substantive feedback and suggestions from participants in usability interviews formed 5 major categories related to (1) the *House* theme and characters, (2) the language and tone of the intervention, (3) the content and features, (4) the audience, and (5) how they might engage with the app. We review each of these themes in detail below, with representative comments from the participants.

#### House Theme and Characters

Most women responded positively to the House theme and look of the characters. In addition to making it more engaging and interesting, women also stated that they thought the characters looked realistic and relatable and that drew them in. A 26-year-old Black woman stated:

I like the characters. I like the words they’ve chosen to use. It’s like, I feel like they’re speaking out to me... I feel like it gets me. Yeah, I like the connection of this app.

U4

A 23-year-old Black woman (U10) stated about the housemother: “she reminded me of myself.” Although most participants expressed positive feedback about the House theme, several noted that for YTW who were not familiar with the house and ball communities, it might be confusing.

#### Language and Tone

Women offered positive feedback regarding the language used. Many participants stated that they liked that the app used terms and phrases they used and that it had a casual, conversational tone. Regarding using slang terms for sexual acts, a 25-year-old Black woman stated:

I actually like that because it’s giving me the real deal...It’s kind of like we all talk, you know...So to actually see this on the app, it makes it more exciting. Like, “Oh, now it’s talking my language.”

U3

A 29-year-old Black woman (U1) stated that the language “made me feel real. It made me feel like I could connect to it.”

Regarding using language that is specific to the transgender community, a participant stated:

I love how they talk about the T a lot. “Girl, watch the T.”

U4

Overall, women shared that the tone and relatable language made them feel comfortable and affirmed. A participant stated:

I haven’t seen an app that was dedicated to us that actually felt like was for us. This actually feels like it’s for us and it actually felt like somebody is listening to us, they’re hearing us, and they want to get our thoughts on how we can better ourselves. They want to help us better ourselves. That’s what it feels like when I was doing the lessons in this app.

U4

Participants shared that they also appreciated that the app talked openly about sex work because it was realistic and would make people who were engaged in sex work feel more comfortable. A 21-year-old person who identifies as gender nonconforming stated:

I like the fact that you all have a client—like the sex workers and stuff.

U7

They went on to state:

I guess that making people feeling more comfortable about talking about stuff like that...You all know what’s really going on and stuff like that.

Regarding an activity related to drug use, participants shared that they thought the tone came across as nonjudgmental and informative. A participant stated:

I thought you were just like informative and educational. You didn’t make it seem like it was bad to do drugs. It’s okay to do them, but you informed us on the effect that it have on the body and stuff like that.

U4

#### Content and Features

In general, women shared that they liked the app because they learned something new. A participant said:

This is just one level that I’m on, and you’ve already hit me somewhere because I’ve already seen three things that I can apply to my everyday life.

U4

Women shared that the content was very important for transgender women. A multiracial 27-year-old woman stated:

I actually think you guys are touching a lot of topics that we don’t, in the community ourselves, don’t talk about sometimes... Just like discrimination period. I don’t see a lot of young people having those conversations... But it’s teaching you that you can overcome it and it’s giving you more positivity and helping you to understand your life personally.

U2

Participants also shared that they liked the features and interactive nature of the app. Participant U2 stated:

It’s actually really interactive and fun. I like it. I could see me playing it.

Participant U4 said:

I really like everything about the app. It’s interesting and I find it fun. It’s like a game I’m playing, but educational. You can play a game, and you can learn at the same time. So, I like it.

Many also shared that they felt motivated by earning stickers and trophies, and they liked how they looked.

Negative feedback about the content included that there was too much text on each page, suggesting that textual content should be edited or broken up into separate pages. They also suggested the inclusion of images of the characters or other images on every page to hold the user’s interest. In addition, several participants suggested that having audio of the housemother’s voice would be more engaging.

#### Audience

Women shared that they thought the app would be helpful for younger transgender women, including women who were still in high school or aged less than 18 years. Participant U9 said:

There are a lot of different things that would have been very, very good for me to know when I was younger that I did not that were in here.

A 24-year-old Black woman (U6) stated that, to her, the app seemed too juvenile, but that it would be good for younger girls. U10 shared that she thought the app would be especially helpful for younger girls who are just transitioning and coming out and are more impressionable. She stated:

I really think it would have a bigger effect from 14 to age 18.

#### App Engagement

Participants in this study only completed portions of the app; several stated that they would do it in chunks, whereas others said they might complete the entire app in one sitting. Participant U2 stated:

I’d do like, four or five [activities] at a time and come back later on, like in an hour and do four or five more.

A 21-year-old multiracial woman (U5) said that she would only spend 30 minutes at a time but would return to it throughout the day. Participant U9 stated:

I’d probably go through the whole thing in one sitting, and then just want to be able to reference things in the future.

## Discussion

### Principal Findings

In summary, in the usability testing of the LifeSkills Mobile app, we found no functional bugs, and all mobile activities deployed as expected. Participant feedback in the usability interviews indicated very good salience of the intervention content for the focal population.

To adapt the intervention, we used multiple methods and informants, including input from substantive experts, transgender women who had previous experience in the delivery of the in-person intervention, and members of the target population. This expertise was critical to reducing the length of the intervention for mobile deployment while also maintaining the core intervention targets, activities, and themes. The success of the development of the initial prototype, with fidelity to the original intervention components, was reflected in the largely positive comments from design session participants, who had relatively few critiques of its content and structure.

The process of building the app, characterized by intensive design sprints with iterative development and testing sprints by members of our advisory group and consultants, led to a bug-free beta version of the app, as demonstrated by the findings of the usability trial. Among testing by 10 YTW, we found no functional bugs and high usability ratings. Substantive input from YTW included improving the clarity of language and reducing the amount of redundancy in 2 activities, which resulted in improved performance.

The LifeSkills Mobile app is characterized by user-centered design features. The advantages of mobile delivery include standardized delivery of content, which increases fidelity, but also flexibility to tailor content to subgroups, giving control of some aspects of content to the user. These features allow more flexibility in the delivery of content than was possible in the in-person group-based intervention.

Taken together, qualitative and quantitative findings suggest that the LifeSkills Mobile app met the usability expectations of the Health-ITUEM [40] for quality, perceived usefulness, perceived ease of use, and user control. In general, feedback from participants in the usability interviews was largely positive and validated the app themes, intervention content, engagement strategies, and language and tone. Perhaps most importantly for engagement and uptake of the app, comments and feedback from semistructured interviews regarding the app activities and content, conducted as part of the usability trial, indicate resonance and salience among YTW. This indicates the success of the app in reflecting the real-life contexts and scenarios faced by YTW. It is important to note that the use of terms and their understanding, particularly slang terms, may differ by group (eg, age, race, and ethnicity) and may also change over time. Thus, it is important to monitor any problems with understanding and relevance and update the app as needed for future use.

In addition, participants reported positive impressions of the gamification approach, and the *House* theme of the app was generally well received. We recognized that this theme might not be applicable or relatable to all YTW. Although a unifying theme, as in the group-based LifeSkills intervention, it is used subtly and mostly symbolically as a tool to communicate the idea of positive mentoring; therefore, direct involvement in or even knowledge of the house and ball communities is generally not necessary to understand its meaning.

LifeSkills Mobile is among the first mHealth interventions that have been developed to meet the specific and unique needs of YTW to reduce sexual risk for HIV acquisition and transmission. It joins other such mHealth initiatives, focusing on transgender women, which are in the development or testing phases [57-60].

### Limitations

The LifeSkills intervention content was developed with and for a largely ethnic minority YTW at high risk for HIV acquisition or transmission; thus, the LifeSkills Mobile app may not appeal to YTW at lower risk. In addition, the app may be particularly effective for younger transwomen, as noted by users. The reasons for this are that the app includes transition-related topics, including access to medical care and tips for safer hormone injections, which are especially important for those who are still in the process of gender transition. However, these tips are important at any age, even after transition, particularly in the context of HIV prevention because of the risk associated with needle use. YTW aged between 16 and 20 years were recruited for this study but were not included in our final sample. The lack of inclusion of YTW aged between 16 and 20 years limits our ability to generalize with confidence to this younger age group. The small sample size limits generalizability. However, the original intervention content, which forms the core of the mobile app, was developed and tested among transgender women aged between 16 and 29 years in 2 previous studies [7,10]. Dynamic app content, including audio, video, and animation, have not yet been added to the app because of resource limitations but are planned for a future iteration. Similarly, the next version of the app will include the implementation of security features, including usernames and passwords, necessary for log-in and browser time-outs. We plan to test the efficacy of LifeSkills Mobile in a future RCT.

### Conclusions

Overall, the adaptation and usability findings indicate that the LifeSkills Mobile app is a highly usable and engaging mobile app to address HIV prevention and related mechanisms of risk among YTW.

## Appendix

Multimedia Appendix 1 Lifeskills mobile phases of app development.

Multimedia Appendix 2 Lifeskills mobile app map.

## Abbreviations

CSQ: Client Satisfaction Questionnaire
Health-ITUES: Health Information Technology Usability Evaluation Scale
mHealth: mobile health
NIH: National Institutes of Health
PEP: postexposure prophylaxis
PrEP: pre-exposure prophylaxis
PSSUQ: Post-Study System Usability Questionnaire
RCT: randomized controlled trial
TasP: Treatment as Prevention
YTW: young transgender women
