# Supplementary figures and images for: A Uniquely Targeted, Mobile App-Based HIV Prevention Intervention for Young Transgender Women: Adaptation and Usability Study
Source: J Med Internet Res. 2021 Mar 31;23(3):e21839. doi: 10.2196/21839 (PMC8047777; doi:10.2196/21839)

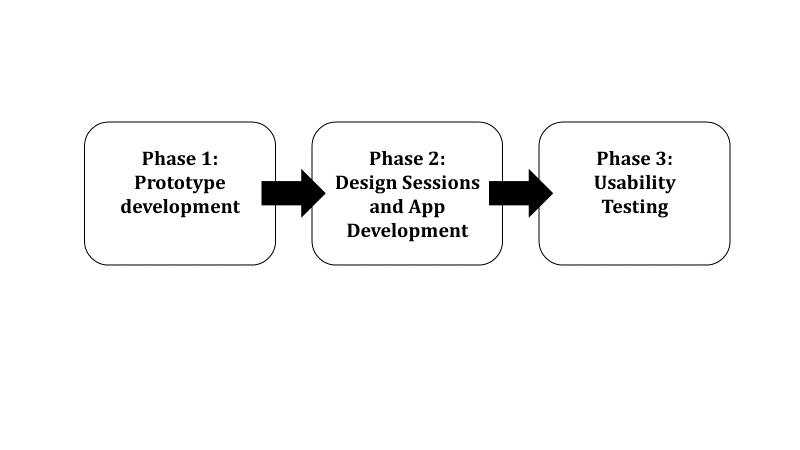

Supplement: Multimedia Appendix 1 [file jmir_v23i3e21839_app1.png]

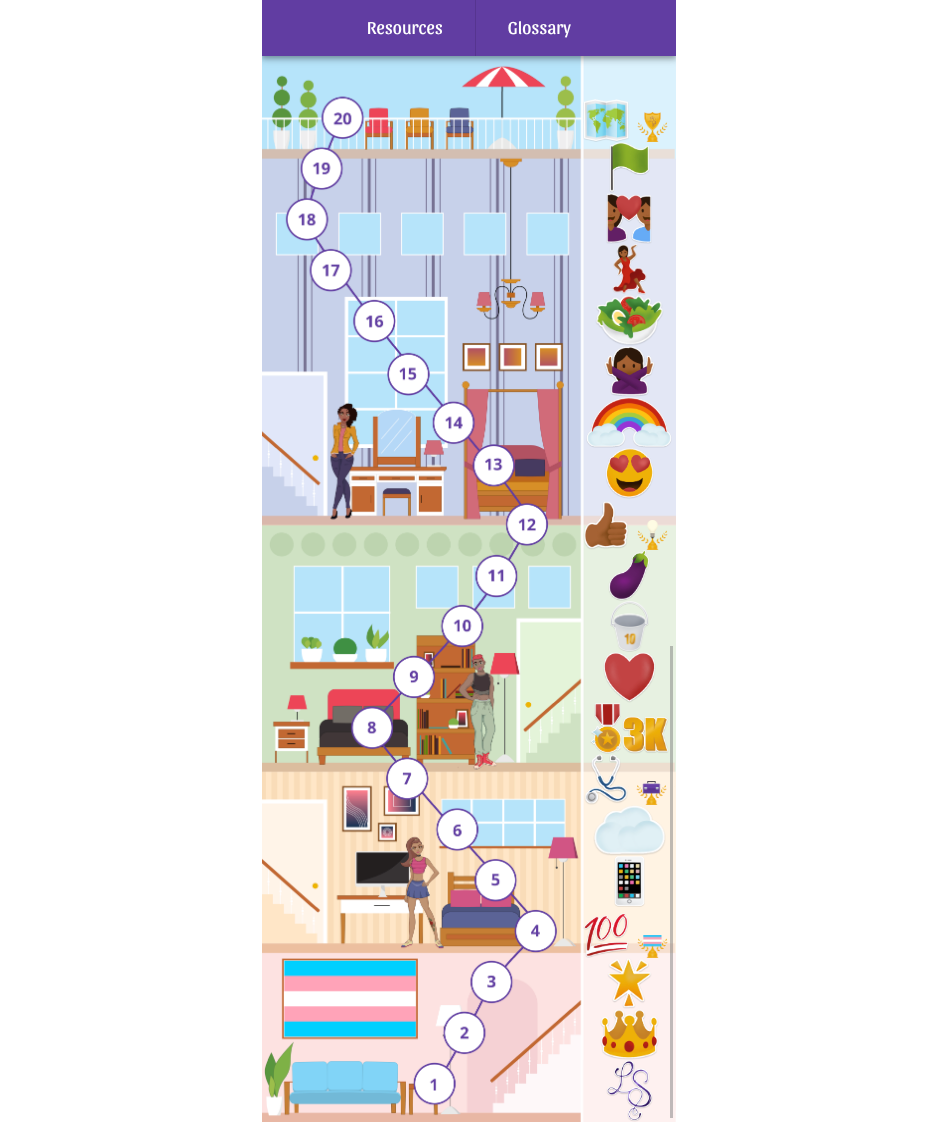

Supplement: Multimedia Appendix 2 [file jmir_v23i3e21839_app2.png]
